# On the Evolution of the Standard Genetic Code: Vestiges of Critical Scale Invariance from the RNA World in Current Prokaryote Genomes

**DOI:** 10.1371/journal.pone.0004340

**Published:** 2009-02-02

**Authors:** Marco V. José, Tzipe Govezensky, José A. García, Juan R. Bobadilla

**Affiliations:** 1 Theoretical Biology Group, Instituto de Investigaciones Biomédicas, Universidad Nacional Autónoma de México, México D.F., México; 2 Department of Preventive and Social Medicine, University of Otago, Dunedin, New Zealand; Tata Institute of Fundamental Research, India

## Abstract

Herein two genetic codes from which the primeval RNA code could have originated the standard genetic code (SGC) are derived. One of them, called extended RNA code type I, consists of all codons of the type RNY (purine-any base-pyrimidine) plus codons obtained by considering the RNA code but in the second (NYR type) and third (YRN type) reading frames. The extended RNA code type II, comprises all codons of the type RNY plus codons that arise from transversions of the RNA code in the first (YNY type) and third (RNR) nucleotide bases. In order to test if putative nucleotide sequences in the RNA World and in both extended RNA codes, share the same scaling and statistical properties to those encountered in current prokaryotes, we used the genomes of four Eubacteria and three Archaeas. For each prokaryote, we obtained their respective genomes obeying the RNA code or the extended RNA codes types I and II. In each case, we estimated the scaling properties of triplet sequences via a renormalization group approach, and we calculated the frequency distributions of distances for each codon. Remarkably, the scaling properties of the distance series of some codons from the RNA code and most codons from both extended RNA codes turned out to be identical or very close to the scaling properties of codons of the SGC. To test for the robustness of these results, we show, via computer simulation experiments, that random mutations of current genomes, at the rates of 10^−10^ per site per year during three billions of years, were not enough for destroying the observed patterns. Therefore, we conclude that most current prokaryotes may still contain relics of the primeval RNA World and that both extended RNA codes may well represent two plausible evolutionary paths between the RNA code and the current SGC.

## Introduction

One of the major transitions in evolution is the origin of the genetic code in the context of the RNA World [1]. The current genetic code is considered to be nearly universal. Given 64 codons and 20 amino acids plus a punctuation mark there are 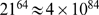 possible genetic codes. Is there something special about the standard genetic code (SGC) that governs all life on Earth? Francis Crick [2] argued that the SGC need not be special at all; it could be nothing more than a “frozen accident”. However, when the SGC was compared to a computer generated random sample of one million alternatives, the natural code emerged as superior in regard to the criterion of polar requirement to every random permutation with a single exception [3]. Recently, statistical estimations with hand-crafted genetic codes analysed *in silico* showed inferior statistical properties (such as information content, scaling and autocorrelation properties) than the SGC [4].

The discovery, about 25 years ago, of RNA molecules with catalytic function [5], [6] led to the notion of an enzyme to include macromolecules composed of either amino acids and/or nucleotides (ribozymes). Naturally occurring ribozymes include groups that excise introns and others that cleave RNA. More recently, the large subunit ribosomal RNA was recognized to be a ribozyme that catalyzes the peptidyl transfer step of protein synthesis [7]. The current paradigm widely accepted is that DNA-based organisms are descendants from simpler RNA-based organisms [2], [8]–[10] strongly supported by developments using synthetic ribozymes [11] and by experiments of evolution *in vitro* of a ribozyme [12]. Presumably, there was an age in the origin of life in which RNA played the role of both genetic material and main agent of catalytic activity (e.g. [2], [9], [13]. For this reason, ribozymes have been proposed as the primeval genetic material [14]. Proteins perform catalysis and DNA stores information, while RNA can do both. This period is known as the RNA World, in which the phenotype was derived from the catalytic properties of RNA and genetic information was only stored on RNA molecules with an RNY pattern [15], where R means purines, Y pyrimidines, and N any of them. Remnants of an RNA ancestry of contemporary organisms seem to be found in their replication machinery, in components of RNA processing and in translational apparatus. Most recently, a New RNA World has been recognized in current organisms, in which the RNA is an integral component of chromosomes and the different types of RNA are key regulatory elements of gene expression (e.g. [16]). The need to change from an RNA- to a DNA-based genome comes from the inherent higher stability of DNA. RNA genomes are now found only in viruses, which require very few genes and, they probably existed prior to the divergence of the three primary kingdoms (Archaea, Eucarya and Prokaryotes) [17]. Extant RNA viruses are possibly remnants of the RNA World. The discovery of retroviruses, which are capable of copying RNA to DNA counterparts, provides a strong lead as to how a DNA takeover may have occurred. Although a great deal of evidence supports the notion of an RNA World, the scenario raises a number of difficult questions. For example, there is an enormous leap from the RNA World to the complexity of DNA replication, protein manufacture and biochemical pathways.

To our knowledge there have not been systematic studies that derive the SGC from the RNA code [2], [18]–[19]. It has been pointed out that this problem is difficult since no intermediate states between the RNA World and the Last Universal Ancestor (LCA) exist [20]. The search for symmetries in the SGC has been made by examining the tRNA [21]–[22], aminoacyl-tRNA synthetases [23]–[26], phylogenetic methods [27]–[28] or by algebraic and geometrical methods [29]–[31].

In a previous work [31], an algebraic and geometrical approach was used to describe the primeval RNA code and to derive from this code the SGC via an Extended RNA code (here called type I). We hypothesized that in order to allow further evolution of the RNA genetic code, the constraints of having an intact message in only one reading frame and/or a fixed RNY pattern have to be relaxed. We consider the notion that translational and transcriptional errors were probably of great importance early in the history of life, when the machinery of protein synthesis was imprecise. Therefore, we considered not a strict comma-less code as proposed by Crick et al. [32] but rather a degenerate RNA code which can be translated in the 1st (RNY), 2nd (NYR), and the 3rd (YRN) reading frames. In this work, we also derive an Extended RNA code (type II) by allowing transversions in the 1st (YNY) and 3rd (RNR) nucleotide bases of the 16 codons of the RNA code. Furthermore, Eigen and Schuster [33] pointed out that it would be advantageous if RNY is symmetric with respect to both plus and minus strand of DNA, so RNY can be complemented by another RNY. This would enable both strands to become equivalent targets for specific recognition enzymes. As was first shown by Arquès and Michel [34], there is indeed a weak form of palindromic sequence or inverse complementarity symmetry in the codon counts in each of the three frames of DNA coding sequences as obtained from large sets of DNA sequences of prokaryotes and eukaryotes. This observation led to the discovery of complementary circular codes [34]–[36] which are related to comma-less codes [37]. They suggested that primeval versions of the genetic code essentially consisted of pairs of sense-antisense codons. Present-day DNA sequences seem to display footprints of this early symmetry, provided the statistics are made over coding sequences issued from groups of organisms and not only from the genome of an individual species [38].

The statistical analysis of DNA sequences has been studied for more than 60 years. Several properties have been unveiled using different methods for their analysis. In particular, the fact that DNA sequences display fractal scaling and the finding that some bacterial chromosomes possess an inverse bilateral symmetry (IBS) was demonstrated by means of a renormalization group (RG) approach [39] and to our knowledge this was the first time that this approach was used for analyzing DNA sequences of whole bacterial genomes [40].

The premise of RG is that in a critical phase transition, the equations describing the system are independent of scale. In this work we have applied the RG transformation in order to look for vestiges of the RNA World and of two putative intermediate states between the RNA World and the LCA.

The notion that present genomes may still retain remnants of their ancestry for more than three billion years has been a subject of controversy [41]–[45].

In this work, we followed the concept that likely a large fraction of genes within a genome are ultimately related by descent to a small number of genes that arose early in our evolutionary history [46]. The rationale of our approach is that if a present day long genome share a vital characteristic of its theoretical shorter earlier self then one knows something about its ancestor, and by extension the common ancestor of its relatives. We contend that by exploiting this approach further and examining genomes closer, one may gain a deeper understanding of our universal ancestor [47] and how the SGC could have originated. Pertinent examples of this approach are the seminal works of Eigen and Winkler-Oswatitsch [48], [49] about the reconstruction of early tRNA and a primordial gene in which they showed that nucleotide sequences existing today still carry, in a hidden form, the patterns of their ancestry.

The article is organized as follows. First, following the criteria of relaxing the RNY code and maintaining the constraint of symmetry between complementary strands [33], we derive two genetic codes from which the primeval RNA code could have originated the SGC. Second, we used genomes of four Eubacteria (*Borrelia burgdorferi*, *Bacillus subtilis*, *Mycoplasma genitalium*, and *Escherichia coli*) and three Archaeas (*Nanoarcheum equitans*, *Methanopyrus kandleri* and *Methanococcus jannaschii*). From each of these organisms, we derived their respective genomes obeying the RNA code, the extended RNA code type I and II. Third, in order to assess if our *in silico* constructed genomes could be biologically meaningful in regard to the different proposed genetic codes, we performed a fractal scaling analysis based upon a renormalization group (RG) technique [39], and we calculated the frequency distributions of distances (FDD) for all their respective codons for all different types of genomes obtained from each of these prokaryotes. Since the concept of fractal scaling and the use of the RG approach may not be familiar to biologists, a brief summary of these subjects are presented and the mathematical aspects of the RG are set out in the Appendix S1.

We show that several scaling and statistical features of the RNA and the extended RNA type I and II codes are still detectable in current prokaryote chromosomes since they show a critical state, with the interesting exception, to some extent, of *M. jannaschii* and *M. kandleri*. In order to test the hypothesis of preservation of patterns, we performed computer simulation experiments in which we allowed for random mutations of current genomes during three billion years. Finally, we reconcile previous debates by concluding that genomes are systems that are constantly under a critical state and therefore, they may show universal properties of scale invariance.

## Methods

### Data sources

The complete sequences of *Borrelia burgderfori*, *Bacillus subtilis*, *Escherichia coli*, *Mycoplasma genitalium*, *Nanoarcheum equitans*, *Methanopyrus kandleri*, and *Methanococcus jannaschii* were retrieved from the NCBI, Genbank resource from the NIH (http:www.ncni.nlm.nih.gov) with the following corresponding accession numbers: **NC_001318**, **NC_000964**, **NC_000913**, **NC_000908**, **NC_005213**, **NC_003551** and **NC_000909**. We used bacterial and archaeal genomes with only open reading frames (ORF), *i.e.* only the protein coding region of the chromosomes. Since our goal was to study statistical properties of whole chromosomes, the original structure of the strands had to be preserved, and therefore we assembled all reported ORF one after the other as originally ordered and oriented in the chromosome (OOO), as if we were reading all ORF in only the leading strand. For example, in the case of *Borrelia burgderfori* the first ORF is located in the leading strand, so it was included without any change to the sequence. However, the second ORF is located on the lagging strand, and if we joined it after the first ORF, we would be mingling both strands. From the flatfile downloaded we got the information about the location of the second ORF (c1796-768), and we added the nucleotides 768 to 1796 of the leader strand to the first ORF. So was done for each ORF. An *ad hoc* program was written in MATLAB in order to create the OOO sequences.

In order to construct the different genomes, we discarded from the OOO genomes the triplets which did not correspond with the desired genetic code by means of an *ad hoc* program written in language Perl. This program permitted to generate the following three different genomes:

- Genomes having only RNY triplets in the first reading frame only, i.e., those that are governed by the RNA code.
- Genomes with only RNY, NYR and YRN triplets, i.e., those that obey an extended RNA code type I.
- Genomes with only RNY, RNR, and YNY triplets, i.e. those that follow an extended RNA code type II.

In order to simplify the description of the results, the first, second and, third types of genomes will be called, here and further, RNA genomes, extended RNA type I genomes, and extended RNA genomes type II, respectively.

For example, if the OOO sequence is: ATG ATA CTA ATG AAG TAT AGT GCT ATT TTA TTA ATA TGT AGC GTT AAT TTA TTT …

Then, the RNA genome is: AGT GCT ATT AGC GTT AAT …

The extended RNA type I genome is: ATG ATA CTA ATG TAT AGT GCT ATT TTA TTA ATA TGT AGC GTT AAT TTA …

The extended RNA type II genome is: ATG ATA ATG AAG TAT AGT GCT ATT ATA TGT AGC GTT AAT TTT …

### Cumulative position plots, distance series, and frequency distributions of distances

Instead of using the classical stochastic random walk mapping rules of DNA, such as the purine-pyrimidine (RY) rule (e. g. [50]), the actual position for a given triplet along the whole genome was determined and from this either a cumulative position plot (CPP) or the actual distance series (distance measured in bases) of that particular triplet were obtained [40]. The FDD for a given triplet was calculated directly from its corresponding distance series along each genome. For example, in the sequence: ATGATACTAATGAAGTATAGTGCTATTTTATTA ATATGTAGCGTTAATTTATTTTGTTTTCAAAATAAATTAACTACTTC… the triplet ATA lies in positions 4, 17, 34, 65, … This series is used for the CPP. The distance series will be 13, 17, 31, … In this way, although we start with a categorical series, we end up with a numerical one.

### Fractal scaling

Most scientists are familiar with the normal or Gaussian distribution, where the moments, such as the mean and variance, have well defined values. In a fractal process, as more data are analyzed, rather than converge to a definite value, the mean continues to increase toward ever larger values or decrease toward ever smaller values. For a fractal process, the moments, such as the arithmetic mean, may not exist. Self-similarity means that the small irregularities at small scales are reproduced as larger irregularities at larger scales. These increasingly larger fluctuations become apparent as additional data are collected. Hence, as additional data are analyzed from a fractal process, these ever larger irregularities increase the measured value of the variance. When the mean is infinite, no natural scale exists by which to gauge measurements. Self-similarity implies that no natural spatial or temporal scale exists, that is, there are events at all scales. Statistical self-similarity implies that a value measured for a property depends on the scale or resolution at which it is measured, and this is called scaling. It has been shown that in DNA sequence analysis, the critical concept is that of scale given the heterogeneity of DNA sequences [40]. The form of the scaling is often a power-law distribution. The fact that the moments of fractal processes may not converge to well-defined values is due to the inverse power law form of fractal distributions. The self-similarity and scaling can be quantitatively measured by the fractal dimension. The fractal dimension tells how many new pieces are resolved as we examine the fractal at finer resolution. Fractals have fractional, noninteger values of dimension, and thus their properties are distinct from those of points, lines, surfaces, and solids that have integer dimensions. The word “fractal” was coined by Mandelbrot [51].

Power-law distributions are known to have statistical self-similarity resulting in the probability density function obeying a functional scaling law which is a renormalization group property. A group is a collection of objects sharing some property in common. The renormalization group relation is the mathematical expression of the underlying process not having a fundamental scale. This lack of a single scale is similarly manifested in the divergence of the central moments.

Classical scaling principles are based on the notion that the underlying process is uniform, filling an interval in a smooth, continuous fashion, and thereby giving rise to finite averages and variances. The new principle is one that can generate richly detailed, heterogeneous, but self-similar structures at all scaling. Thus, length, area, and volume are not absolute properties of the system but are functions of the units of measure [52].

### Renormalization group

Now we present the basic ideas of the RG approach in order to illustrate how this procedure can be applied for analyzing the genome of a prokaryote. Briefly, the renormalization group (RG) analysis, introduced in field theory and in critical phase transitions, is a very general mathematical and conceptual tool, which allows one to decompose the problem of finding the macroscopic behavior of a large number of interacting parts into a succession of simpler problems with a decreasing number of interacting parts, whose effective properties vary with the scale of observation [39]. The RG permits to determine the scaling properties of a system. At the outset, a set of equations that may describe the behavior of the system is assumed. Then the length scale at which the system is being described is changed. By moving away from the system, some of the details are lost. At the new scale, the same set of equations is applied, but possibly with different coefficients. The objective is to relate the set of equations at one scale to the set of equations at the other scale. In this way, the scaling properties of the system can be obtained. The RG approach deals with the concept that a critical point results from the aggregate response of an ensemble of elements. The basis of RG is that exactly at a second order phase transition, a critical state, the equations describing the system are independent of scale and details become irrelevant.

The concept of RG is useful for systems that exhibit the properties of scale invariance and self-similarities of the observables at the critical point of the system [53]. The two main transformations of RG are decimation and rescaling. When we go from the fine scale to the coarse scale, the process is called decimation. The idea of RG is to decimate the degrees of freedom, while rescaling so as to keep the same scale by calculating, for example, the relative dispersion (the ratio of the standard deviation to the mean). The procedure can be repeated using groupings of two, three, four and more data points. In this way the fractal dimension that is independent of the degree of coarse-graining can be determined.

All figures related to the RG operation were obtained by aggregating one by one the distance series and the length of the final distance series was dependent upon the length of the distance series. The whole collection of plots regarding the CPPs, the scaling analysis, and the frequency distributions of triplets in all types of genomes of the seven chosen prokaryotes, are available upon request.

### Estimating the fractal dimension from RG

Methods for assessing the fractal characteristics of time-varying or distance-varying signals are useful for DNA sequences analysis. DNA sequences which vary, apparently irregularly, have been considered to be driven by external influences which are random. The Hurst exponent 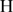 gives a measure of the smoothness of a fractal object, with 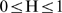. A low 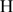 indicates a high degree of roughness, so much that the object almost fills the next-higher dimension; a high 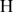 indicates maximal smoothness so that the object intrudes very little into the next-higher dimension. A flat surface has Euclidean dimension 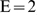; a slightly roughened surface with, for example, a fractal dimension 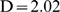 intrudes only a little into three-dimensional space. The general relationship is [52]:
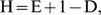



The exponent 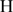 is calculated from the slope of the log-log relationship between the ratio of the range to the standard deviation and the length of the interval observed. The descriptor “rescaled analysis” means that dividing by the standard deviation for each binning is a method of normalization allowing one range to be compared to another.

Mandelbrot [51], [54] calls 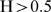 persistence because it means that increases are more likely to be followed by increases at all distances, and 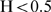 antipersistence because increases are more likely to be followed be decreases at all distances. Thus, 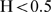 signals are considerable more irregular than those with 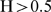. If the fractal dimension is given by 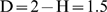, then the data points are linearly independent of one another and this would be the case for a slope of 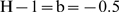 in the log-log plots of 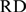 versus the aggregation number, 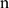. This fractal dimension corresponds to an uncorrelated random process with normal statistics, often referred to as Brownian motion. If 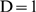, then the irregularities in the distance series are uniform at all distances. A fractal dimension of unity implies that the distance series is regular, such as it would be for simple periodic motion. Most distance series of triplets have fractal dimensions that fall somewhere between the two extremes of Brownian motion with 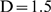, and complete regularity with 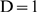, a range which is indicative of long-range correlations, also called fractional Brownian motion [51].

In each case we determined the scaling properties of the distance series of triplets and their reverse complementary triplets via a renormalization group (RG) approach from which the Hurst exponent 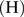 and hence, the fractal dimension 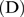 can be estimated.

## Results

### The RNA code and its two evolutionary pathways: the extended RNA codes type I and II

**Figure 1:**
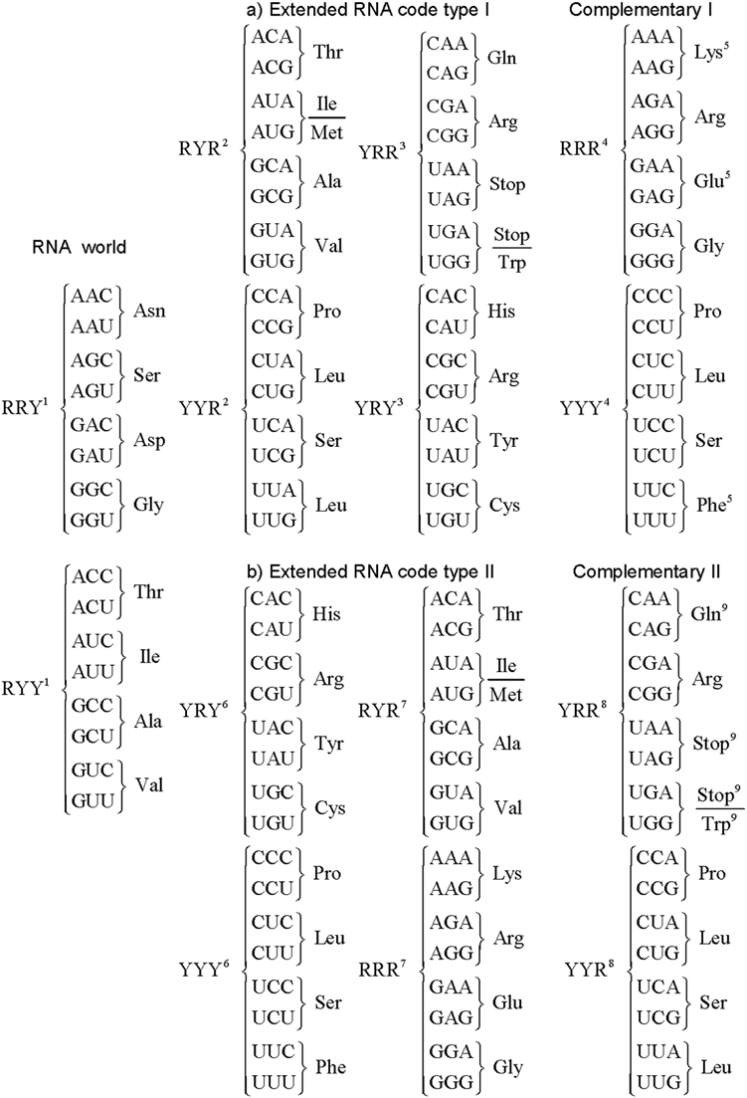
1. Triplets that appear in the 1st reading frame and that are of the type RNY; a) The extended RNA code type I: 2. Triplets that appear in the 2nd reading frame of the RNY pattern and are of the type NYR; 3. Triplets that appear in the 3rd reading frame of the RNY pattern and are of the type YRN; 4. Triplets that do not appear in the extended RNA code type I (RRR or YYY); 5. Amino acids that do not belong to the extended RNA code type I; b) The extended RNA code type II: 6. Triplets that underwent a mutation in their 1st nucleotide position (YRY and YYY); 7. Triplets that underwent a mutation in their 3rd nucleotide position (RRR and RYR); 8. Triplets that do not appear in the extended RNA code type II (YYR and YRR). 9. Amino acids that do not belong to the extended RNA code type II.

The RNA World code consists of only RNY codons that comprises 16 out of the 64 possible triplets and which codify for 8 amino acids. To wit, RNY codons are AAY (Asn), AGY (Ser), ACY (Thr), AUY (Ile), GAY (Asp), GGY (Gly), GCY (Ala), and GUY (Val) (see Fig. 1, 1st column).

Natural selection does not generate novelties from scratch. It works on what already exists; either transforming a system to give it new functions or combining several systems to produce a more elaborate one. It innovates with what it has at hand and this process has been dubbed as the evolutionary tinker [55]. Since the primitive translational apparatus may have been imperfect, shifts in the reading frames probably occurred. Assuming a primordial sequence of the type …RNYRNYRNY…, frame-shift reading mistranslations in the 2nd and 3rd reading frames produced sequences of the type …NYRNYR…, and …YRNYRN…, respectively. In other words, codons of the type NYR and YRN emerged. *The 3 sets of codons*, *RNY*, *NYR*, *and YRN are here defined as the codons of the extended RNA code type I* (Fig. 1a, 1st upper 2nd and 3rd columns). This extended code includes 48 triplets which specify 17 amino acids, including AUG, the start codon, and the 3 known stop codons. The remaining 16 codons which are of the type RRR and YYY (Fig. 1a, upper 4th column), can be obtained by mutations and/or by frame-shift readings of the extended RNA code type I, and we defined them as the complementary code of the extended RNA code type I. This code is to be compared with the circular code proposed by Arquès and Michel [34], in which there are 20 triplets in each reading frame and the codons AAA, TTT, GGG, and CCC, which belong to the union of the sets RRR and YYY, are excluded.

Note that given the RNA code, frame-reading mistranslations conferred obvious evolutionary advantages, since in fact, by allowing reading slippages, 32 new triplets (which codify for 9 new amino acids) and 3 stop triplets, emerge. Konecny et al. [19] first noted that by allowing reading slippages there were at least two hidden messages in the RNY code which are AUG and CAU that are encountered in the 2nd and 3rd reading frames, respectively.

Now, if we allow for transversions in the RNA code in the 1st or the 3rd nucleotide bases, codons of the type YNY and RNR arise. We define the extended RNA code type II as consisting of the 3 sets of codons RNY, RNR, and YNY (Fig. 1b, 1st, lower 2nd and 3rd columns). This code comprises 48 codons that specify 18 out of the 20 amino acids but no stop codons. The remaining 16 codons which are of the type YYR and YRR (Fig. 1b, lower 4th column), can be obtained by mutations and/or by frame-shift readings of the extended RNA code type II, and we defined them as the complementary code of the extended RNA code type II. Interestingly, this code is in agreement with the observation that the order of triplet frequencies RNY>RNR>YNY>YNR is a general attribute of coding sequences [56]. These authors proposed that this order may reflect the evolution of the genetic code from an RNY structure, providing a comma-free readout via wobble-intermediates to the present form [56]. The extended RNA code type I has also been derived according to algebraic and geometrical considerations from the RNA code [31]. In particular, it has been shown that the 16 codons in each of the three subsets of the extended RNA code type I can be represented by a symmetrical 4-dimensional hypercube [31]. Analogously, in a forthcoming paper, we show that the 16 codons in each of the four subsets (including its complementary) of the extended RNA code type II can also be represented each by symmetrical 4-dimensional hypercubes. Note that the NYR and YRN codons are required to be added simultaneously to the RNA code, and so do the YNY and RNR codons, in order to generate the extended RNA code type I and II, respectively. In this manner, we ensure that both extended RNA codes type I and II comply with the criterion of the symmetry constraint of complementary strands. This criterion is consistent with other works in which the requirement of complementarity in tRNAs and aminoacyl-tRNA-synthetases renders almost perfect symmetry of the table of the genetic code (e. g. [25]–[26]). Therefore, we are proposing two intermediate processes, not mutually exclusive, by which the RNA code could have originated the SGC.

### Fractal scaling analysis

It has been proved that in order to study the statistical properties of DNA sequences in bacterial chromosomes it may be necessary to consider both halves of the chromosome because they can display inverse bilateral symmetry (IBS) [37], or bilateral symmetry (BS) as it is found in this work. The IBS can be reflected by the fact that the statistical properties of a distance series of a given codon in one half of the prokaryote chromosome are similar to the corresponding distance series of its reverse complementary codon in the other half [37].

**Figure 2:**
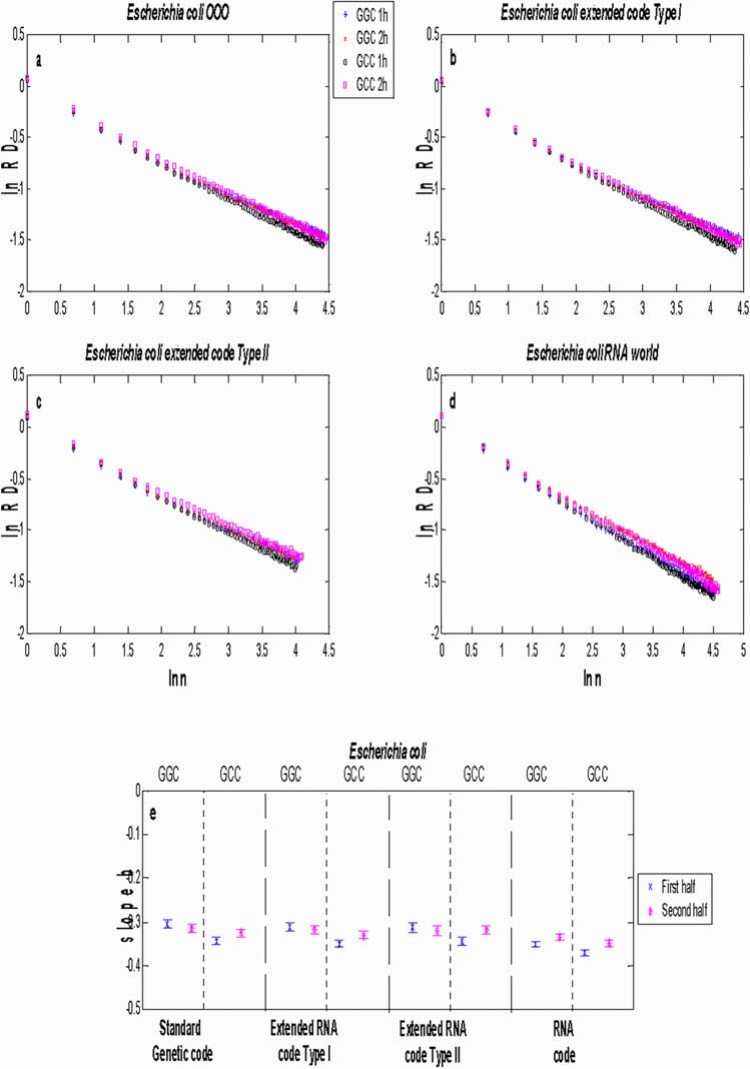
Scaling aggregation analysis of a given codon in the 1st and 2nd halves of each genome of *E. coli*. In each plot of Fig. 2(a–d), the corresponding 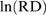 as a function of 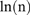 for GCC and GGC in a) the OOO genome; b) the extended RNA code type I, c) the extended RNA code type II, and in d) the RNA World. The estimates of the slope 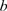 together with their confidence intervals for each codon in each of the different genomes are illustrated in Fig. 2(e). The symbols 1 h and 2 h stand for the 1st and 2nd halves of the genome, respectively.

We present results for *E. coli*, *M. genitalium*, *N. equitans* and *M. kandleri* in Figs. 2(a–e)–[Fig pone-0004340-g003] 4(a–e), respectively. The aggregation analysis for a given codon and its reverse complementary in the 1st and 2nd halves of the different genomes (current, both extended RNA genomes and the RNA genome) show a linear decay of the 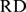 as the size of the window 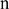 increased in a log-log plot, as it is shown in Figs. 2(a–d)–[Fig pone-0004340-g003] [Fig pone-0004340-g004] 5(a–d). It is observed that the apparent variation diminishes as the number of data points in each binning period is enlarged. These straight lines indicate that the distance series for all triplets in all analysed genomes are random monofractals. This means that perusing the variability of a triplet in any small segment, if stretched out, will look much like the whole, i.e., they are self-similar. It also means that 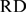 changes do not have a typical scale like in a Gaussian distribution. The estimates of the slope 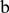 together with their confidence intervals (at ±3 sd) for some codons in each of the different genomes of *E. coli*, *M. genitalium*, *N. equitans* and *M. kandleri* are illustrated in Figs. 2(e)–[Fig pone-0004340-g003] [Fig pone-0004340-g004] 5(e), respectively. The range of estimates of the slope 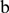 for *E. coli* for the triplets GGC and GCC, in the 1st half of the genomes, goes from 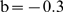 to 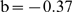 (hence 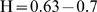 and 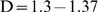). For the 2nd half of the genomes, the range of 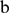 goes from 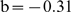 to 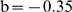 (hence 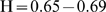 and 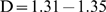). The range of estimates of the slope 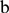 for *M. genitalium* for the triplets AAU and AUU, in the 1st half of the genomes, lies between 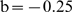 and 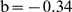 (hence 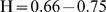 and 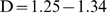). For the 2nd half of the genomes, the range of 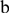 goes from 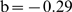 to 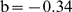 (hence 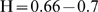 and 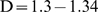). All values of 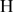 are greater than 0.5, a fact which indicates long-range correlations. In the case of *B. burgdorderi* and *B. subtilis*, slightly more than 50% of the scaling exponents of a given triplet in one half of the genome are statistically similar to its corresponding reverse complementary triplet in the other half of the genome, a fact which clearly indicates that their chromosomes display IBS (not shown). However, in the case of *E. coli*, *M. genitalium*, and the three Archaeas, the type of symmetry of each chromosome is in general difficult to discern. In the case of *E. coli* (see Fig. 2(e)), it is apparent that the scaling exponents of GGC in the 1st and 2nd halves of the extended RNA genomes type I and II, but not in the RNA genome, are statistically similar to the scaling exponents of GCC in the 2nd and 1st halves of the genomes, respectively. Thus, there is IBS between GGC and GCC in these latter genomes. Bilateral symmetry can be observed in regard to GGC in all genomes since the scaling exponent of this triplet in the 1st half of each genome is statistically similar to the exponent of this same triplet in the 2nd half of each genome. The greatest disparities are observed when a codon of the type RRR or YYY in an extended RNA genome type I, or a codon of the type YRR or YYR in an extended RNA genome type II, are compared with the original prokaryote genome (Table 1). In the case of *M. genitalium* there is IBS in regard to AAU and AUU in the original genome since the scaling exponent of the former in the 1st half of the original genome is similar to the scaling exponent of the latter in the 2nd half of the genome. IBS is also observed for AAU and AUU in the extended RNA genomes type I and II, and in the RNA genome, since the magnitude of the scaling exponent of AAU in the 1st (or in the 2nd) half is similar to the estimate of the scaling exponent of AUU in the 2nd (or in the 1st) half. Note that the magnitude of the scaling exponents for the reverse complementary triplets AGC and GCU in *M. kandleri*, are not preserved. For *M. kandleri* and *M. genitalium*, the aggregation analysis deviates from straight lines, which means that there is an inverse power-law decorated by a dominant log-periodic modulation. Despite all these plethora of behaviors, it is noteworthy that the scaling exponent values obtained for most codons and its complementary in the original genomes of all organisms analysed turned out to be similar or very close to the ones obtained with their respective genomes governed by any of the extended RNA codes and even with the RNA code.

**Figure 3 pone-0004340-g003:**
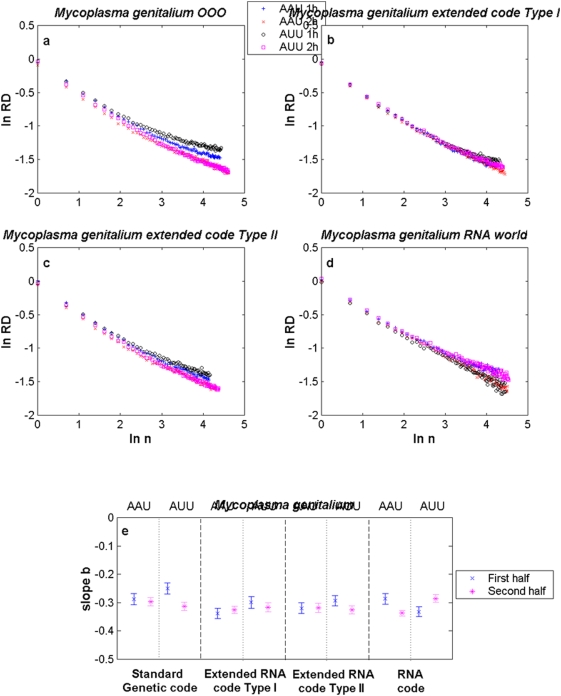
Scaling aggregation analysis of a given codon in the 1st and 2nd halves of each genome of *M. genitalium*. In each plot of Fig. 2(a–d), the 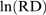 as a function of 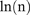 for AAU and AUU in a) the OOO genome; b) the extended RNA code type I, c) the extended RNA code type II, and d) the RNA World. The estimates of the slope 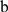 together with their confidence intervals for each codon in each of the different genomes are illustrated in Fig. 2.2(e). The symbols 1 h and 2 h stand for the 1st and 2nd halves of the genome, respectively.

**Figure 4 pone-0004340-g004:**
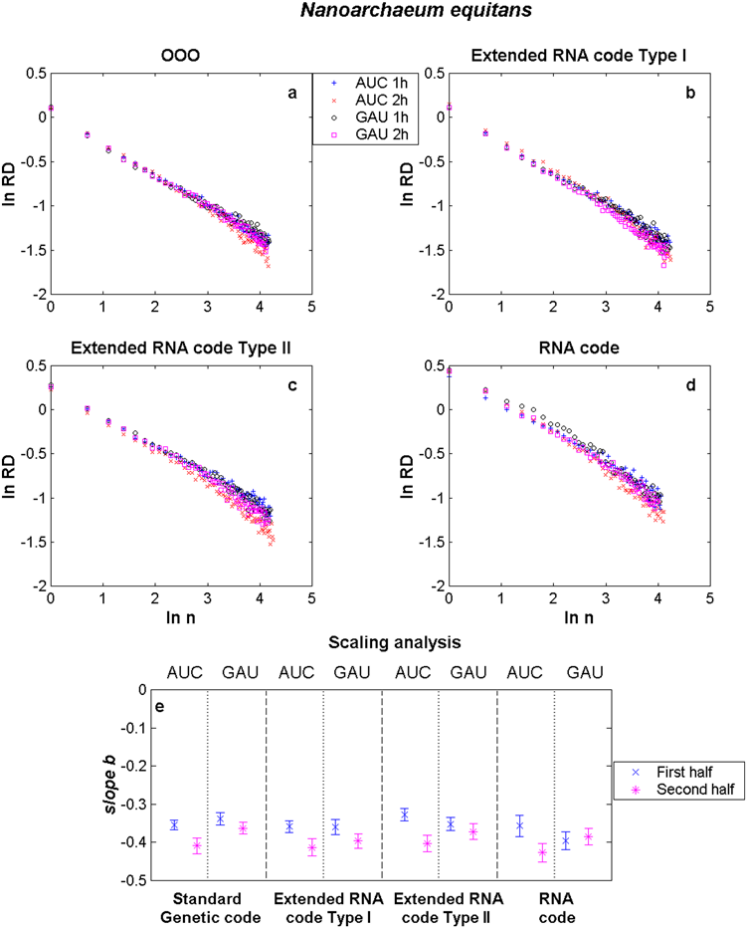
Scaling aggregation analysis of a given codon in the 1st and 2nd halves of each genome of *N. equitans*. In each plot of Fig. 2.3(a–d), the 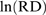 as a function of 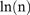 for AAU and AUU in a) the OOO genome; b) the extended RNA code type I, c) the extended RNA code type II, and d) the RNA World. The estimates of the slope 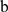 together with their confidence intervals for each codon in each of the different genomes are illustrated in Fig. 2.3(e). The symbols 1 h and 2 h stand for the 1st and 2nd halves of the genome, respectively.

**Figure 5:**
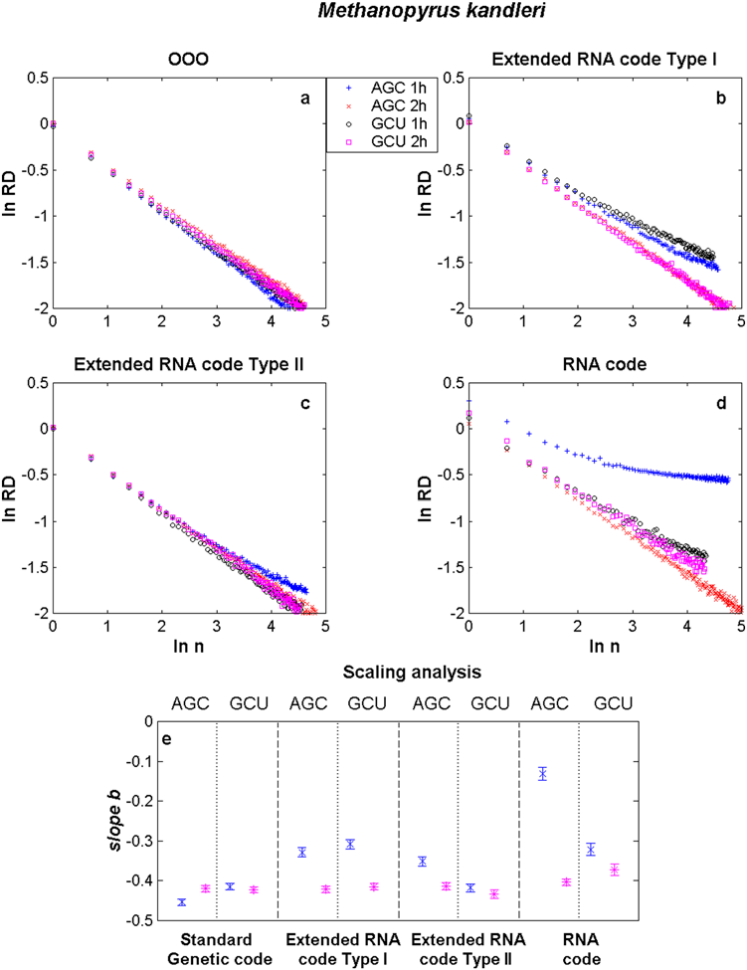
Scaling aggregation analysis of a given codon in the 1st and 2nd halves of each genome of *M. kandleri*. In each plot of Fig. 2.4(a–d), the 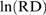 as a function of 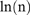 for AGC and GCU in a) the OOO genome; b) the extended RNA code type I, c) the extended RNA code type II, and d) the RNA World. The estimates of the slope 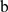 together with their confidence intervals for each codon in each of the different genomes are illustrated in Fig. 2.4(e). The symbols 1 h and 2 h stand for the 1st and 2nd halves of the genome, respectively.

**Table 1:** Codons of the different genomes that show critical scale invariance.

	Codon type Chromosome half	RNA world	
		RRY-RYY	
		1^st^	2^nd^
Archaea	*N. equitans*	8	12
	*M. kandleri*	2	5
	*M. jannaschii*	4	8
Eubacteria	*B. subtilis*	5	8
	*B. burgdorferi*	3	6
	*E. coli*	4	4
	*M. genitalium*	7	8

	Codon type Chromosome half	Extended RNA code type I						Complementary code		Total	
		RRY-RYY		RYR-YRY		YYR-YRR		RRR-YYY			
		1^st^	2^nd^	1^st^	2^nd^	1^st^	2^nd^	1^st^	2^nd^	1^st^	2^nd^
Archaea	*N. equitans*	12	11	13	11	13	14	3	2	41	38
	*M. kandleri*	6	13	11	13	10	7	6	2	33	35
	*M. jannaschii*	6	7	10	8	5	5	0	0	21	20
Eubacteria	*B. subtilis*	10	14	13	12	9	10	11	10	43	46
	*B. burgdorferi*	12	12	15	12	7	9	2	8	36	41
	*E. coli*	9	8	13	14	10	7	2	3	34	32
	*M. genitalium*	10	12	12	13	12	11	10	8	44	44

	Codons type Chromosome half	Extended RNA code type II						Complementary code		Total	
		RRY-RYY		RYR-YRY		RRR-YYY		YYR-YRR			
		1^st^	2^nd^	1^st^	2^nd^	1^st^	2^nd^	1^st^	2^nd^	1^st^	2^nd^
Archaea	*N. equitans*	11	13	14	13	14	15	11	13	50	54
	*M. kandleri*	7	11	8	10	15	7	5	5	35	33
	*M. jannaschii*	11	12	12	9	16	15	7	11	46	47
Eubacteria	*B. subtilis*	9	10	12	8	12	9	8	3	41	30
	*B. burgdorferi*	8	12	12	9	11	14	3	7	34	42
	*E. coli*	10	7	9	13	13	10	4	4	36	34
	*M. genitalium*	10	11	10	11	16	14	8	10	44	46

In Table 1, we summarize the results of the scaling analysis of all codons for each genome in the seven selected organisms. For each prokaryote, we report how many codons, in each half of each genome, have scaling exponents which are statistically indistinguishable from its corresponding halves of its current genome. For example, there are 12 out of the 16 codons in the 2nd half of the RNA genome which show scale invariance (the slopes are statistically undistinguishable) in regard to the current genome of *N. equitans*. Similarly, there are only 2 out of the 16 codons in the 1st half of the RNA genome which display scale invariance in comparison with the current genome of *M. kandleri*. Note that the codons NYR and YRN that belong to the extended RNA code type I as well as the codons RNR and YNY that pertain to the extended RNA code type II share the RYR and YRY codons. In both extended codes there are more RNY codons exhibiting scale invariance than their corresponding RNA genomes of the RNA world. This is true for all prokaryotes except in the case of the 2nd half of the RNA genome of *M. jannaschii* in which there are 8 codons in comparison with its corresponding 2nd half of the extended RNA genome type I in which there are 7 codons. The number of RYR-YRY codons whose scaling exponents are statistically equal to those of the current genomes ranges from 8 to 15 out of the 16 codons that belong to any of the extended RNA codes. Note that scale invariance is less frequent in codons RRR-YYY, that comprise the complementary code of the extended RNA code type I, than the ones found in the extended RNA code type II. These types of codons do not show scale invariance in the extended RNA code type I in the genome of *M. jannaschii*, but all 16 codons of this type exhibit scale invariance in the extended RNA code type II. Conversely, the number of codons YYR-YRR, which constitute the complementary code of the extended RNA code type II, that display scale invariance, are in general less than the ones encountered in the extended RNA code type I. The exception to this rule is again *M. jannaschii*. Overall, the total number of codons that exhibit scale invariance of the extended RNA genomes ranges from 20 (*M. jannaschii*) to 54 (*N. equitans*) out of the 64 codons of the SGC.

### Frequency distributions of distances

**Figure 6:**
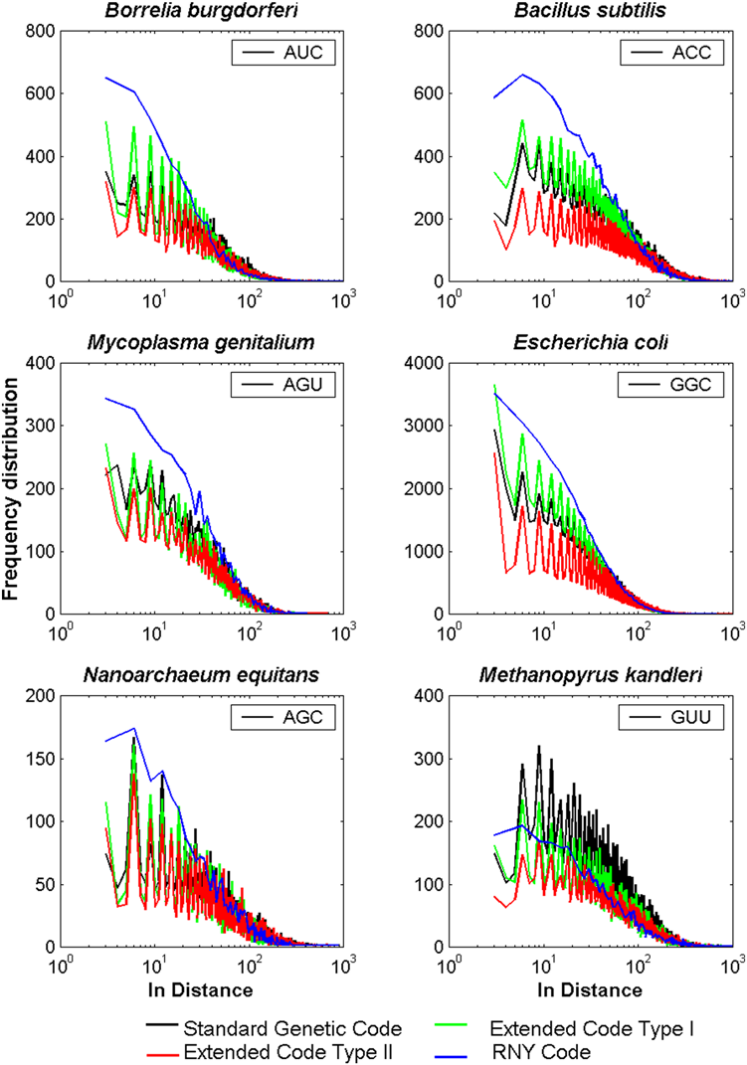
Log-linear plots of the FDD of a given triplet in each genome of a) *B. burgdorferi* b) *B. subtilis* c) *M. genitalium* d) *E. coli* e) *N. equitans* f) *M. kandleri*. The different colors indicate the SGC (black) of the OOO genomes, the extended RNA code type I (green) and II (red), and the RNA code (blue).

It is well known that coding and non-coding sequences display different statistical and evolutionary properties (e. g. [49], [57]–[60]). One of them refers to the fact that in coding sequences there is a 3-base periodicity in the nucleotide sequences as reflected by all possible correlations of single bases, duplets and triplets [49], [42], [61], the FDD of triplets [62], the autocorrelation function [63], [59] or by the mutual information function of single bases [64]. We remark that all these methods, implicitly or explicitly, or perhaps inadvertently used the three overlapping reading frames when a given count of a triplet was made. Shepherd [41]–[43] suggested that the first code was composed of repeating sequences of coding triplets having the form RNY, that these have been replaced by the present SGC, but that vestiges of these repeating sequences are still detectable. In Fig. 6 the FDD's of some codons of four bacteria and two Archaeas are shown. Only one triplet for each organism was selected since the corresponding FDD of its complementary triplet is very similar along any entire genome. When IBS is present, the FDD of a given triplet in the 1st half of the genome is practically indistinguishable from the corresponding distribution of its reverse complementary triplet in the 2nd half (not shown). The FDD for each of the 16 codons of the RNY genomes showed a power-law fractal behavior with no oscillatory behavior because RNY codons appear only in the 1st reading frame in a RNY sequence as mentioned earlier. This is in contrast with the rhythmic oscillatory decaying pattern every 3 bases of the distributions of triplets of the original prokaryote genomes and the genomes of the extended RNA type I and II. Note the similar behavior of the FDD for any codon of the extended RNA genomes type I and II to the ones observed in current prokaryote genomes. It is noteworthy to mention that, albeit 16 codons have been discarded from the current genomes in order to obtain both extended RNA genomes, the phase of each of the FDD's remains unaltered. In particular, the FDD of the extended RNA code type I are very similar to the current genomes of *B. subtillus*, *M. genitalium* and *E. coli*, whereas the corresponding distributions in the extended RNA code type II are similar to the current genome of *B. burgdorferi*. The oscillatory decaying pattern with 3-base periodicity of the FDD can clearly be ascribed to the fact that the count of a given triplet is carried out by considering its three reading frames. But this condition is not necessary, since in the case of the FDD of RRR or YYY codons that appear only in the 2nd and 3rd reading frames of the extended RNA code type I, also exhibit an oscillatory decaying pattern every 3 bases (not shown). The same holds true for the YRR and YYR codons that pertain to the complementary code of the extended RNA code type II.

### Perturbation analysis

Herein, we examine the robustness of the estimates of the scaling exponents in order to see if not all information of ancestral organisms has been erased at least for the last 3 billions years of evolution. Additionally, the effect of this perturbation upon the CPP's and FDD's was also examined.

**Figure 7:**
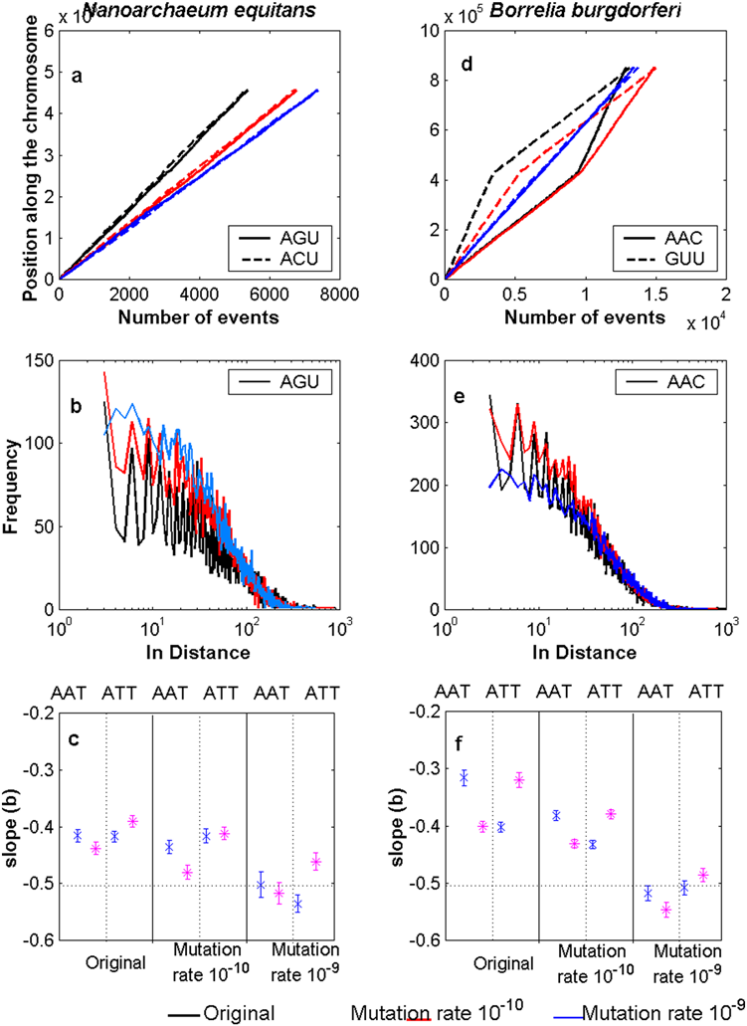
Robustness of current genomes. In a) CPPs (triplets AGU-ACU), b) FDD (triplet AGU), and c) the estimates of the slope 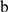 together with their confidence intervals for the triplets AAT-ATT for *N. equitans*. In d) CPPs (triplets AAC-GUU), e) FDD (triplet AAC), and f) the estimates of the slope 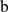 together with their confidence intervals for the triplets AAT-ATT for *B. burgdorferi*. Intact genomes (plots in black) were subjected to computer simulation experiments of random mutations at a rate of 10^−10^ (plots in red) or 10^−9^ (plots in blue) per site per year during three billions of years.

An ad hoc program in Matlab was elaborated to perform the simulations. Two different mutation rates were considered: 10^−10^ and 10^−9^ mutations per site per year. The number of point mutation occurring in one million years per chromosome 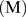 was calculated using the Poisson distribution taking into account the chromosome length. 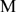 sites along the chromosome were chosen using a discrete uniform distribution. In each of these sites the respective nucleotide was substituted by selecting at random (with equal probabilities) one of the four possible nucleotides. The procedure was repeated 3000 times in order to simulate 3×10^9^ years. The simulated experiments were made blindly regardless of synonymous or non-synonymous mutations, transitions or transversions, or the specific codon usage of each organism. Results for *N. equitans* and *B. burgdorferi* are shown in Fig. 6. Note that at 10^−10^ the CPPs, the FDD's are practically unaltered for both organisms whereas for a rate of 10^−9^ clear deviations from the current patterns start to emerge. Likewise, at 10^−10^ the estimates of the corresponding scaling exponents still reflect long-range correlations whereas for a rate of 10^−9^ the exponents tend to indicate random behavior (Fig. 7(c, f)). We remark that at the rate 10^−10^ during three billions years, one third of the whole genome is mutated.

## Discussion

We have derived two plausible evolutionary paths of the different prokaryote genomes since the RNA world to current prokaryote genomes via the extended RNA code type I and type II. We argue that the life forms that probably obeyed the extended RNA code type I and/or type II were intermediate between the ribo-organisms of the RNA World and the cenancestor of Bacteria, Archea, and Eucarya. Our results support the notion that evolution did not erase all vestiges typical of the RNA World in today prokaryote genomes, not only in terms of an enrichment of RNY codons, but also revealing the existence of an underlying ancient fractal structure.

The primeval RNA code consists of 16 codons that specify 8 amino acids (then this code shows a slight degeneration), but considering the 2nd and 3rd reading frames there are 48 codons that specify 17 amino acids and the three stop codons. If transversions in the 1st or 3rd nucleotide bases of the RNY pattern are permitted, then there are also 48 codons that encode for 18 amino acids but no stop codons.

An outstanding result regarding the scaling properties of the distance series of some codons from the RNA genomes and most codons of the extended RNA genomes type I and II, is that they turned out to be identical or very close to those of the present genomes. Since the magnitudes of the scaling exponent of the distance series of these codons are unaltered, both in evolutionary time and in actual distance series, we can say that they show critical scale invariance according to the renormalization group theory.

In this work we have used a renormalization group approach to obtain an expression for the aggregated relative dispersion. The aggregated relative dispersion indicates that the process has a preferential scale length, 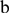, in addition to the monofractal behavior determined by the inverse power-law index 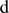. Thus there is the interleaving of two mechanisms, one that is scale free and produces the monofractal, and the other has equal weighting on a logarithmic scale and is sufficiently slow as to not disrupt the much faster fractal behavior. The tying together of the long and the short distance scales is necessary in order to adaptively regulate the complex DNA or RNA sequences in a changing environment. The log-periodic modulation of the inverse power-law is a consequence of the correlation function satisfying a renormalization group relation and having a complex fractal dimension [53]. Sornette argues that the log-periodicity is a result of what he calls Discrete Scale Invariance, that is, also a consequence of renormalization group properties of the system. In the present work we have provided the basis for a fractal relationship in DNA sequences showing correlations extended over longer distances than would ordinarily be expected under the hypothesis of uncorrelated and independent random signals. Near the critical scaling the system has fractal properties, which means that there are spatial correlations at all length scales. Therefore, the nature of prokaryote genomes pertains to a class of phenomena, where events at many scales of length make contributions of equal importance. Any scaling analysis of DNA sequences must take into account the entire spectrum of length scales since we are facing a system near its critical point [39]. The resulting RNA genomes as well as the extended RNA type I and II genomes, as obtained from different prokaryotes are of relevance not only for studying the origin of life but also for generating minimal genomes subject to Darwinian evolution.

The fact that the RNA genomes of *M. kandleri* and *M. jannaschii* depart from the RNA genomes of other prokaryotes rules out the possibility that the scale invariance is due to the self-similarity of the DNA sequences or that they are due to artifacts of the RG technique.

The FDD of the RNA genomes never show an oscillatory pattern every 3 bases, as was proposed some years ago by Shepherd [41]–[43]. Instead, the remaining genomes show a rhythmic oscillatory decaying pattern every 3 bases. In other words, Shepherd's patterns are found only in both extended RNA codes type I and II, when compared with current bacterial genomes. But this fact does not contradicts Shepherd's observation since he himself stated that “…*counts of certain correlated base combinations are only given at separation intervals of three bases and are zero at all other separations (i.e. the correlation signal amplitude is a maximum and the background is zero)*”. Shepherd estimated that the time of last use of the comma-less code appears to be of the order of 3 billion years ago [65]. Despite this long time there seems to remain original vestiges surviving in current DNA prokaryote sequences. Additionally, the oscillatory behavior is not due to the degeneracy of the genetic code, as was suggested by Herzel and Groβe [64], since the RNA code is degenerate but their corresponding FDD of triplets hardly oscillate. Rather the oscillatory behavior is attributable to the fact that a triplet can appear in two or three reading frames.

Our perturbation analysis suggest that critical scale invariance was selected since the RNA world and that an enrichment of RNY codons in the genes for proteins could be a primitive remnant due to this fact and is not necessarily due to codon usage [44] In any case, the RNA code was not all erased by mutations. It may also well be that the excess of RNY codons is principally attributable to preference for the corresponding tRNAs (e.g. [2]). Both extended RNA codes offer a clue about a chronological order in which the different amino acids might have appeared in clusters due to the joint addition of the codons. Three steps of appearance are considered: The amino acids encoded by RNY triplets are assumed to appear first; amino acids encoded by the triplets NYR and YRN or by RNR and YNY appeared in a second stage; amino acids of the type RRR and YYY or YYR and YRR appeared in the last step. This order is to be compared with a consensus temporal order of amino acids, based upon 60 different criteria, as derived by Trifonov [66]. Our proposed temporal order of amino acids can be regarded as another criterion for a consensus chronological order. In both extended RNA codes, most RNY triplets are in rough agreement with the consensus order. The temporal order of amino acids based upon the extended code type II seems to have more hits with the Trifonov's consensus order.

In summary, the attractive and appealing idea of fossilized remnants of the primeval and the extended RNA codons in today existing genes is given credit in this work.

## Supporting Information

Appendix S1 Mathematical aspects of the scaling analysis.
(0.10 MB DOC)
