# Supplementary material for: On the Evolution of the Standard Genetic Code: Vestiges of Critical Scale Invariance from the RNA World in Current Prokaryote Genomes
Source: PLoS One. 2009 Feb 2;4(2):e4340. doi: 10.1371/journal.pone.0004340 (PMC2631149; doi:10.1371/journal.pone.0004340)
Supplement: Appendix S1 — Mathematical aspects of the scaling analysis. (0.10 MB DOC) [file pone.0004340.s001.doc]

### **Supporting Information**

# Appendix S1 Mathematical aspects of the scaling analysis.

# Scaling analysis: Hurst exponent and fractal dimension

The purpose of RG is to translate in mathematical language the concept that the sum is the aggregation of an ensemble of arbitrarily defined sub-sums, each sub-sum defined by the sum of sub-sums and so on. In other words, the RG approach implies that a critical point results from the aggregate response of an ensemble of elements. Let us assume the renormalization group scaling relation:

$\mathrm{F}(\mathrm{x})=\frac{\mathrm{F}(\mathrm{bx})}{\mathrm{a}}$ (1)

This relation expresses the property of $\mathrm{F}(\mathrm{x})$ being self-affine, i.e., the graph of $\mathrm{F}(\mathrm{x})$ on a scale $\mathrm{bx}$ has to be scale down by a factor $1/\mathrm{a}$ to obtain the desired function on scale $\mathrm{x}$. Whereas self-similarity refers to the fact that the shapes are identical under magnification, self-affinity expresses the fact that $\mathrm{F}$ and $\mathrm{x}$ have to be scaled by different amounts for the two views to become identical. Scale invariance means reproducing itself on a different time or space scale.

An observable $\mathrm{F}$ which depends on a control parameter $\mathrm{x}$ is scale invariance under the arbitrary change $\mathrm{x}\to\mathrm{bx}$ if there is a number $\mathrm{a}(\mathrm{b})$ such that Equation (1) holds. The solution of (1) is:

$\mathrm{F}(\mathrm{x})=\mathrm{cx}^{\mathrm{d}}$ with $\mathrm{d}=\frac{\ln(\mathrm{a})}{\ln(\mathrm{b})}$ (2)

Power-laws are the hallmark of scale invariance as the ratio, $\frac{\mathrm{F}(\mathrm{xb})}{\mathrm{F}(\mathrm{x})}=\mathrm{b}^{\mathrm{d}}$, does not depend on $\mathrm{x}$, i. e. the relative value of the observable at two different scales depends simply on the ratio of the two scales. Considering the solution in (2) we get:

$$\frac{\mathrm{b}^{\mathrm{d}}}{\mathrm{a}}=1=e^{i2\pi\mathrm{n}}$$

This leads to:

$\mathrm{d}=\frac{\ln(\mathrm{a})}{\ln(\mathrm{b})}+i\frac{2\pi\mathrm{n}}{\ln(\mathrm{b})}$, (3)

which characterizes the system in terms of complex fractal dimensions. The imaginary part of the fractal dimension translates itself into a log-periodic modulation decorating the leading power law behavior. This has been dubbed Discrete Scale Invariance by Sornette [53].

Now let us examine how the relative dispersion $(\mathrm{RD})$ changes as a function of the number of adjacent data elements we aggregate. We start by aggregating $\mathrm{n}-$adjacent data points, so that the $\mathrm{j}-$element in such an aggregation is given by $\mathrm{Y}_{\mathrm{j}}^{(\mathrm{n})}=\mathrm{Y}_{\mathrm{nj}}+\mathrm{Y}_{\mathrm{n}(\mathrm{j}-1)}+\mathrm{Y}_{\mathrm{n}(\mathrm{j}-2)}+...+\mathrm{Y}_{\mathrm{n}(\mathrm{j}-(\mathrm{n}-1))}$. In terms of these new data the average is defined as the sum over the total number of data points, i.e.

$\overline{\mathrm{Y}}^{(\mathrm{n})}=\frac{1}{\left[\mathrm{N}/\mathrm{n}\right]}\sum_{\mathrm{j}=1}^{\left[\mathrm{N}/\mathrm{n}\right]} \mathrm{Y}_{\mathrm{j}=1}^{(\mathrm{n})}\mathrm{Y}_{\mathrm{j}}^{(\mathrm{n})}=\mathrm{n}\overline{\mathrm{Y}}^{(1)}$, (4)

where the bracket denotes the closest integer value, and $\mathrm{N}$ is the original number of data points. The variance for a monofractal time series is similarly given by [52]:

$\mathrm{Var}(\overline{\mathrm{Y}}^{(\mathrm{n})})=\mathrm{n}^{2\mathrm{H}}\mathrm{Var}(\overline{\mathrm{Y}}^{(1)})$, (5)

where $\mathrm{H}$ is the Hurst exponent, and the superscript on the average variable indicates that it was determined using all the original data without aggregation and the superscript on the average variable indicates that it was determined using the aggregation of data points. Thus, $\mathrm{RD}$ for an aggregated data set is:

$\mathrm{RD}^{(\mathrm{n})}=\frac{\sqrt{\mathrm{Var}(\overline{\mathrm{Y}}^{(\mathrm{n})})}}{\overline{\mathrm{Y}}^{(\mathrm{n})}}=\frac{\sqrt{\mathrm{n}^{2\mathrm{H}}\mathrm{Var}(\overline{\mathrm{Y}}^{(1)})}}{\mathrm{n}\overline{\mathrm{Y}}^{(1)}}=\mathrm{n}^{\mathrm{H}-1}\mathrm{RD}^{(1)}$. (6)

which is exactly an inverse power-law in regard to the aggregation number for the Hurst exponent in the interval $0\leq\mathrm{H}\leq1$.

Equation (6) has the form of a renormalization group (RG) transformation, where the underlying phenomenon is dominated by a single scaling exponent and, hence it represents a monofractal. It is well established [52, 67], that the exponent in such scaling equations is related to the fractal dimension, $\mathrm{D}$, of the underlying time series by $\mathrm{D}=2-\mathrm{H}$. A simple monofractal time series, therefore, satisfies the inverse power-law relation for the $\mathrm{RD}$ given by Equation (6) which can be expressed by the linear regression relation [67, 68]:

$\ln\mathrm{RD}^{(\mathrm{n})}=\ln(\mathrm{a})+\mathrm{bln} (\mathrm{n})$, (7)

where $\mathrm{b}=1-\mathrm{D}$.
